# Alveolar soft part sarcoma of the right calf: A case report

**DOI:** 10.1097/MD.0000000000018952

**Published:** 2020-01-31

**Authors:** Bin Wang, Huanhuan Wang, Jinlong Wei, Limei Qu, Lingbin Meng, Ying Xin, Xin Jiang

**Affiliations:** a Department of Radiation Oncology; b Department of Pathology, The First Hospital of Jilin University, Changchun, China; c Department of Internal Medicine, Florida Hospital, Orlando, Florida; d Key Laboratory of Pathobiology, Ministry of Education, Jilin University, Changchun, China.

**Keywords:** alveolar soft part sarcoma, excision, myogenic determination factor 1, radiation therapy, transcription factor E3

## Abstract

**Rationale:**

Alveolar soft part sarcoma (ASPS) is a rare malignant soft tissue neoplasm with controversial histogenesis. ASPS accounts for 0.5% to 1% of all soft tissue sarcomas. Because of its rarity, ASPS is easily misdiagnosed, increasing the risk of incorrect treatment.

**Patient concerns:**

A 6-year-old female patient presented with a history of a 2.0 × 2.5 × 3.0-cm mass in the deep soft tissues of her right lower extremity.

**Diagnoses:**

Histopathological features indicated the diagnosis of ASPS. Microscopically, a diffuse arrangement of tumor cells or pseudoalveolar architectures separated by thin and well-vascularized fibrous septa were observed. Immunohistochemical staining of the tumor cells indicated positivity for transcription factor E3, myogenic determination factor 1, and periodic acid–Schiff–diastase (PAS-D) and showed a Ki-67 proliferating index of approximately 20%.

**Interventions:**

The patient underwent enlarged resection of the tumor and was treated with radiotherapy.

**Outcomes:**

During the 3-year follow-up, the patient has remained in good condition, with no symptom recurrence, distant metastatic spread, or significant toxicity during or after treatment. The patient remains under regular surveillance.

**Lessons:**

Its low incidence, lack of characteristic clinical manifestations, and atypical location often lead to ASPS misdiagnosis and subsequent incorrect treatment. Nuclear expression of transcription factor E3 is of diagnostic value for ASPS. At present, there is no consensus on the treatment for ASPS. In-depth pathological analysis is needed to better understand the characteristics of this tumor.

## 1 Introduction

Alveolar soft part sarcoma (ASPS) is a rare malignant soft tissue neoplasm with an unclear histogenesis that occurs in 0.5% to 1% of all soft tissue sarcoma.^[1]^ Clinically, it often presents as a soft, non-ulcerated, painless, and slow-growing mass with a high potential to metastasize.^[2]^ ASPS commonly affects the muscle and deep soft tissue of the extremities, trunk, and head and neck of adult patients and mainly involves the head and neck and rarely the extremities of children.^[3,4]^ Here we present a case report of a 6-year-old female patient diagnosed with ASPS in the soft tissue of her right low extremity, including the clinical manifestations, pathological characteristics, immunophenotype, molecular genetic features, differential diagnosis, and treatment.

## 2 Case presentation

**Figure 1:**
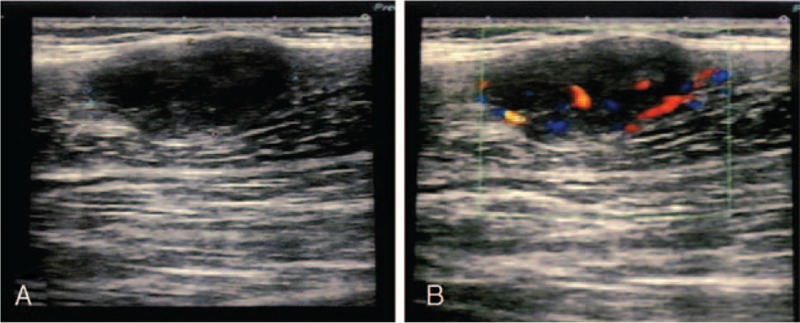
Ultrasonography features of alveolar soft part sarcoma. (A) B-scan ultrasonography revealed a hypoechoic, homogeneous soft tissue mass with clear boundary, which measured 22.7 mm in diameter. (B) Color Doppler flow imaging revealed marked increased vascularity within the mass.

**Figure 2:**
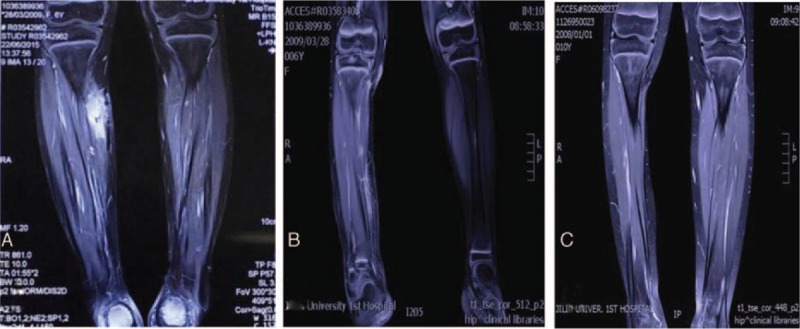
Histopathological features of ASPS. (A) The characteristic pseudoalveolar pattern of ASPS (H&E staining, original magnifications ×200). (B) Eosinophilic cytoplasmic granules can be seen in some tumor cells (H&E staining, original magnifications ×400). (C) Acid–Schiff–diastase-positive granules were seen in intracellular cytoplasm of tumor cells (immunohistochemical staining, magnification ×400). (D) A very low Ki-67 index was obtained (immunohistochemical staining, magnification ×200). (E) The tumor cells are strongly nuclear positive for myogenic determination factor 1 (immunohistochemical staining, magnification ×200). (F) The tumor cells show strong nuclear positivity to TFE3, confirming the presence of the *TFE3* gene at Xp11 translocation with the *ASPL* gene at 17q25 creating an ASPL-TFE3 fusion protein (immunohistochemical staining, magnification ×200). ASPS = alveolar soft part sarcoma, H&E = hematoxylin and eosin, TFE3 = transcription factor E3.

**Figure 3:**
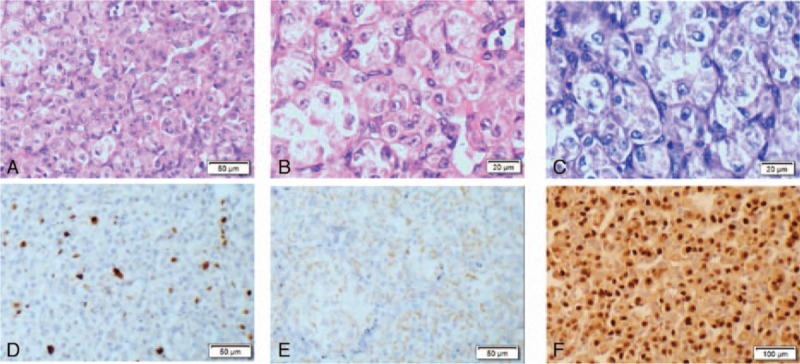
Magnetic resonance imagings (MRI) of the calf. (A) The MRI of the calf before second enlarged resection. (B) The MRI of the calf after radiotherapy. (C) The MRI of the calf at 3-year after radiotherapy.

A 6-year-old female patient presented to our hospital with a history of a 2.0 × 2.5 × 3.0-cm mass in the deep soft tissues of her right lower extremity. She denied any other associated symptoms including pain, obvious swelling, and local fever. A mass 2.3 cm in diameter in the deep soft tissues of her right lower extremity was visible by color Doppler ultrasound (Fig. 1A). This feature was suggestive of capillary hemangioma (Fig. 1B). An excision biopsy was performed on June 3, 2015. During surgery, a clearly circumscribed tumor without obvious capsule was observed. Biopsy revealed ASPS measuring approximately 2.5 × 1.5 × 1.3 cm; tumor thrombolysis was observed in blood vessels and almost all margins were positive. Microscopically, the tumor cells showed a diffuse arrangement of cells or pseudoalveolar architectures separated by thin, well-vascularized fibrous septa (Fig. 2A). The tumor cells exhibited pleomorphism with oval to polygonal shape, with abundant cytoplasmic granules and eosinophilic deposits with irregular nuclei (Fig. 2B). Immunohistochemically, the tumor cells were positive for transcription factor E3 (TFE3) (Fig. 2F) (which confirmed the histological diagnosis of ASPS), myogenic determination factor 1 (MyoD1) (Fig. 2E), and (focal) PAS-D (Fig. 2C), with negativity for desmin and synaptophysin. The Ki-67 proliferating index was about 20% (Fig. 2D). Postoperative magnetic resonance imaging (MRI) revealed abnormal signals in the right calf measuring approximately 1.4 × 0.83.2 cm. T1 imaging showed a low signal whereas T2 imaging showed a slightly higher signal intensity. Tumor parenchyma was markedly enhanced after enhancement scanning (Fig. 3A). Other parts of the patient, including her chest and abdomen, showed no abnormalities. A second enlarged resection was performed on June 24, 2015. Twenty days after the operation, the patient was treated with three-dimensional conformal radiation therapy. Under the Varian linear accelerator, 6 MV X-ray external irradiation was applied to the area of the tumor bed. The total dose was 5000 cGy at 200 cGy each time, 5 times a week. The patient developed no adverse reactions to radiotherapy or any abnormal blood indicators, except grade 1 skin toxicity in the radiation field. MRI after radiotherapy showed longer T1 and T2 abnormal signals without marked enhancement consistent with postoperative changes, accompanied by exudation of the surrounding soft tissue (Fig. 3B).

Routine follow-up has been performed since the end of radiotherapy, and the patient has been followed-up for 3 years by MRI examination of the right lower extremity. The MRI performed on July 10, 2018, revealed no local tumor recurrence (Fig. 3C). No chronic toxic side effects, such as limited joint activity, and no evidence of distant metastatic spread were observed. The patient remains under regular surveillance.

## 3 Discussion

ASPS is a rare and potentially aggressive malignant soft tissue neoplasm with an uncertain histogenesis according to the 2013 WHO classification of soft tissue tumors.^[5]^ In 1952, Christopherson et al^[6]^ first defined ASPS as an entity in a series of 12 cases. The site of origin remains controversial, with both myogenic and neurogenic origin proposed.^[7,8]^ ASPS accounts for 0.5% to 1% of all soft tissue sarcomas.^[1]^ The age of onset, location, size, and metastatic status of the tumor are correlated with prognosis. The overall mean survival time is 7 years and the 2-, 5-, and 10-year survival rates of patients are approximately 87%, 62%, and 43%, respectively.^[9]^ ASPS primarily affects young patients, with a peak incidence between 15 and 35 years of age.^[10]^ There is a slight preponderance in female patients, who account for 60% of all cases.^[11]^ In adults, ASPS often occurs in the deep soft tissues of the extremities and torso, especially the upper and lower extremities.^[3]^ However, the head and neck are the common lesion sites in children.^[3]^ Although ASPS has a relative lack of symptoms and often presents as a painless and slowly growing mass, it is a vascularized neoplasm with a tendency for vascular invasion and hematogenous distant metastases. The frequency of metastasis increases with patient age at diagnosis.^[12]^ The lungs are the most frequent site of metastasis, followed by the bones and brain.^[13]^

Clinically, ASPS typically presents as a soft, non-ulcerated, indolent, and slow-growing mass.^[2]^ The imaging feature of ASPS includes a hypervascular mass on ultrasonography, computed tomography (CT), and MRI. Ultrasonography of ASPS often reveals a slightly hypoechoic or hyperechoic soft tissue mass with markedly increased blood flow.^[14]^ On CT images, ASPS appears as a soft tissue mass with rich vascularity, which is homogeneous and isodense to muscle with vigorous enhancement. ASPS exhibits hypervascular lesions on contrast-enhanced CT or angiography, with dense tumor staining and tortuous, dilated draining veins.^[15]^ ASPS has characteristic MRI findings. T1WI shows equal or slightly higher signals whereas T2WI shows an uneven high signal. Moreover, large numbers of thick and tortuous blood vessels are observed around the tumor. Due to the rapid blood flow passing through vessels, it presents as a flowing void effect on MRI. This is a relatively characteristic feature of alveolar soft tissue sarcoma. Multiple peritumoral and intratumoral tortuous signal voids and intense enhancement are visible in enhancement scanning.^[15]^ Despite the many imaging methods for diagnosis of this disease, unfortunately, the patient in our case only underwent a preoperative ultrasound examination without CT or MRI.

ASPS has unique histopathological features. The tumor shows typical organ-like or pseudoalveolar architectures separated by thin, well-vascularized fibrous septa. Clear or abundant eosinophilic granular cytoplasm in the tumors may be observed in PAS staining. The abundance of blood vessels causes frequent intravasation of tumor cells, which is correlated with the high metastatic potential.^[16,17]^ Specific and immunohistochemical staining play significant supporting roles in ASPS diagnosis. In immunohistochemistry, tumor cells partly express cytoplasmic MyoD1. MyoD1 is not expressed in the nucleus; 40% of cases are desmin-positive and 20% to 30% cases are focally vimentin-positive. Although tumor cells are usually negative for epithelial markers, they may be positive for muscle-derived markers.^[12]^ Genetically, only nuclear expression of TFE3 is of diagnostic value. Ladanyi et al^[18]^ first detected the ASPL-TFE3 fusion gene mMRA in ASPS tissues by reverse transcription-polymerase chain reaction. ASPS is caused by an unbalanced translocation, namely der(17)t(X:17)(p11;p25), which fuses the *TFE3* gene at Xp11 to the *ASPL* gene at 17q25, creating the ASPL-TFE3 fusion protein. ASPS is easily misdiagnosed because of its low incidence, lack of characteristic clinical manifestations, and nontypical location, which can lead to incorrect therapy.

As with other soft tissue tumors, complete surgical or extensive local excision are the primary treatment options for this disease.^[19]^ The status of the surgical margin is a significant prognostic factor, indicating the presence of active tumor tissue.^[20]^ Marker et al^[21]^ suggested that normal tissue 1.0 to 1.5 cm outside the tumor boundary should be removed to ensure a negative incision margin. Because of the positive surgical margins in the present case, the patient underwent an enlarged resection after excision biopsy. The benefit of adjuvant therapy in ASPS remains controversial.^[22]^ Sherman et al^[23]^ suggested that radiation therapy played a beneficial role in enhancing local control after limited resection, which may reduce the risk of brain metastasis.^[24]^ In the present case, the patient received adjuvant three-dimensional conformal radiation therapy to the tumor bed at a dose of 45 Gy. No studies have reported significant survival benefits in patients undergoing adjuvant chemotherapy after surgery. However, recent reports demonstrated satisfactory clinical outcomes following the use of angiogenesis inhibitors to treat metastatic ASPS.^[25]^ However, without metastasis, the patient in the present case was not eligible for antiangiogenesis therapy.

## 4 Conclusion

ASPS mainly involves the head and neck in children. We present a rare case report of a 6-year-old female patient diagnosed with ASPS in the soft tissue of her right calf who underwent successful radiotherapy and achieved a complete response after undergoing a second enlarged resection. A good therapeutic effect was observed. No symptom recurrence, distant metastatic spread, or significant toxicity occurred during or after the treatment. The treatment is still well-tolerated by the patient, who is currently being followed-up. ASPS is easily misdiagnosed because of its low incidence, lack of characteristic clinical manifestations, and nontypical locations, which can lead to incorrect therapy. At present, there is no consensus on the treatment of ASPS. In-depth pathological analysis is needed to better understand the characteristics of this tumor.

## Acknowledgments

We would like to thank Editage (www.editage.cn) for English language editing.

## Author contributions

**Funding acquisition:** Ying Xin.

**Investigation:** Huanhuan Wang, Lingbin Meng.

**Resources:** Jinlong Wei.

**Visualization:** Limei Qu.

**Writing – original draft:** Bin Wang.

**Writing – review & editing:** Xin Jiang.
